# Initial assessment of a species-specific rebound measurement mode for non-human primates

**DOI:** 10.1186/s12917-026-05578-6

**Published:** 2026-05-25

**Authors:** Yingbo Liu, Yunhao Su, Kaiming Zhang, Ji Fu

**Affiliations:** 1 The School of Mechanical Engineering, Zhejiang Sci-Tech University, Hangzhou, China; 2 Deze Preclinical Research Laboratory Corporation, Xuancheng, Anhui China

**Keywords:** Non-human primates, Intraocular pressure, Rebound tonometry, Bland-Altman analysis, iFalcon™ V100

## Abstract

**Background:**

Accurate measurement of intraocular pressure in non-human primates is of significant importance in veterinary clinical practice, particularly for the prevention, detection, and management of glaucoma in non-human primates. However, no veterinary tonometer is currently designed specifically for non-human primates.

**Objective:**

To investigate the accuracy of the macaque-specific measurement mode of the iFalcon™ V100 rebound tonometer.

**Methods:**

IOP was increased stepwise from 5 mmHg to 90 mmHg in 3–5 mmHg increments. At each target pressure, the system was stabilized for 10 s, then the iFalcon™ V100 recorded six consecutive rebound readings and their average. Each step yielded 2–3 such average values. The results were compared with a high-precision invasive pressure sensor. Linear regression and Bland-Altman analyses were performed, and the measurement range was stratified (5–30, 30–60, 60–90 mmHg) for refined assessment.

**Results:**

The iFalcon™ V100 showed strong positive correlation with the invasive sensor (y = 0.99x + 0.42, R² = 0.99). The mean difference was − 0.036 mmHg (95% limits of agreement: -3.747 to 3.675 mmHg). Within the 5–30 mmHg range, agreement was excellent (mean difference: -0.063 mmHg). Acceptable agreement was maintained up to 90 mmHg.

**Conclusions:**

This ex vivo study provides an initial assessment of the iFalcon™ V100 rebound tonometer in non-human primate eyes. The device showed good correlation and agreement with an invasive pressure sensor across a wide pressure range. While the sample size was limited, these preliminary findings support the potential of this species-specific measurement mode for IOP assessment in non-human primate research. Further studies with more eyes and in vivo conditions are needed to confirm its reliability and repeatability.

## Introduction

Intraocular pressure (IOP) is defined as the tissue pressure resulting from the fluid and other contents inside the eye [1]. IOP is one of the core physiological parameters for the diagnosis and monitoring of ophthalmic diseases, most notably glaucoma. It serves as a key clinical indicator for assessing glaucoma risk and guiding treatment decisions. Elevated IOP is the most important modifiable risk factor for glaucoma, and its association with disease prevalence has been well established in epidemiological studies [2, 3].

Tonometry is the method of indirectly measuring IOP by detecting a physical parameter (e.g., probe rebound speed or corneal indentation) and converting it to IOP using a built-in algorithm [4]. Current clinical and research practices commonly employ several tonometry methods, such as indentation tonometry [5, 6], applanation tonometry [4, 5], non-contact tonometry (air-puff tonometry) [4, 5], pneumotonometry, and rebound tonometry [5, 7]. Among the various tonometry methods, rebound tonometry is one of the most frequently used instruments in practice [5]. Its key benefits include the elimination of an air-puff, no requirement for topical anesthesia, as well as convenient operation and portability [8]. Meanwhile, it is practically painless for the subject under examination [9]. Rebound tonometry’s small probe size lends itself well to applications involving animal models [10].

Historically, other tonometry techniques have also been applied to non-human primates (NHPs), though each carries significant limitations. Applanation tonometry has been used in macaques [11, 12] but requires topical anesthesia and may cause corneal abrasions with repeated use. Indentation tonometry was employed in early NHPs studies [13] but is highly operator-dependent and inaccurate in non-human corneas. Non-contact (air-puff) tonometry avoids corneal contact but is sensitive to alignment and often requires heavy sedation [14]. Pneumotonometry has rarely been reported in NHPs due to difficulty in placing the scleral ring [15]. These methods lack species-specific calibration and their accuracy in NHPs remains questionable.

Although rebound tonometry has been extensively studied and validated in clinical human medicine, numerous research teams have also applied this technique to IOP measurement in various animal species, including rodents [16, 17], rabbits [18], dogs [19] and NHPs [11]. Ocular anatomical features—such as corneal thickness, curvature, and biomechanical properties—vary considerably across species [20]. Consequently, applying a calibration mode designed for one species (e.g., dogs) to another species (e.g., cats) can lead to substantial measurement errors, highlighting the necessity of species-specific calibration protocols [20].

Among animal species requiring species-specific calibration, NHPs warrant particular attention due to their evolutionary proximity to humans [21]. NHPs share highly similar ocular anatomy with humans [22], positioning them as a valuable reference platform for validating and calibrating rebound tonometry techniques and for advancing glaucoma research. Therefore, the systematic measurement and study of IOP in macaque eyes using rebound tonometry are of significant importance in veterinary ophthalmic research, particularly in the field of glaucoma. Moreover, findings from NHPs studies can inform and refine IOP measurement algorithms for both human and other animal applications. To our knowledge, however, no rebound tonometer currently offers an independent measurement mode specifically designed for NHPs, representing a critical gap that warrants systematic investigation.

To address this gap, a newly developed device—the iFalcon™ V100—has recently been introduced. However, no dedicated studies have yet demonstrated its accuracy. Therefore, the present study aimed to validate its measurement precision using an ex vivo macaque eye model to systematically evaluate the performance of the iFalcon™ V100 in measuring IOP, using measurements from a high-precision invasive pressure sensor as the reference standard within a pressure range of 5–90 mmHg.

## Ethical review

This study used two fresh eyes from the same experimental rhesus macaque (*Macaca mulatta*): a 12-year-old male with no documented ocular abnormalities. It should be noted that these eyes were obtained from experimental macaque that was euthanized for reasons unrelated to this study; no live animals were used, nor was any direct euthanasia performed for the purposes of this research. The animal was naïve, and no experimental procedures had been performed on it prior to euthanasia. The eyes were not used for any other studies. After the death of the experimental macaques, the eyes were cleaned with sterile saline and placed in sterile saline-filled reagent bottles for transport on ice. Experiments were completed within 8 h of enucleation to preserve the viability of the ocular tissues as much as possible, prevent tissue degradation, and minimize potential effects on the experimental results.

## Materials and methods

### Study design

This study was designed as an ex vivo methodological validation study for IOP measurement. No experimental grouping, intervention groups, or control groups were established, as the objective was to evaluate measurement agreement between a rebound tonometer and an invasive reference pressure sensor under controlled pressure conditions. The experimental design followed a controlled pressurization protocol using a saline infusion system to generate IOP across a defined range (5–90 mmHg), enabling direct comparison between tonometric measurements and invasive sensor reference values.

### Instruments

The instruments used in this study included the following:

- A custom-designed 3D fixation platform (support bowl)
- An iFalcon™ V100 rebound tonometer (Hangzhou iFalcon Co., Ltd., a pre-production prototype equipped with a measurement algorithm specifically designed for NHPs).Custom mounting bracket of iFalcon™ V100
- A set of disposable infusion tubing and a saline drip bottle
- Two experimental macaque eyes
- An invasive pressure sensor enclosed within an 18G needle (protected temperature-sensitive pressure sensor, VSensor, Shanghai Xinyuan Electronic Technology Co., Ltd., factory-calibrated. The invasive pressure sensor was pre-loaded into the lumen of an 18G needle. The needle served as a protective sheath to prevent mechanical damage during insertion. Cyanoacrylate adhesive was applied to the needle hub to seal the lumen and secure the sensor, forming a sensor-needle assembly. The sensing element remained exposed at the bevel opening, ensuring direct contact with intraocular fluid without extending beyond the needle tip.)
- A 24G syringe needle, an 18G syringe needle, absorbent paper, and cyanoacrylate tissue adhesive.

### Experimental procedures

The experimental method in this study was partially based on the approach described by Kapeller et al. [23], but with certain modifications. Kapeller et al. elevated IOP by raising the fluid bag and puncturing the anterior chamber of the eye. In contrast, in this study, pressurization was achieved by puncturing the vitreous cavity. Fluid infused into the vitreous cavity transmitted pressure to the anterior chamber, allowing IOP measurement by the rebound tonometer. Because the anterior chamber space is very limited, placing both the pressure sensor needle and the infusion needle there would have restricted the achievable pressure range and caused corneal deformation. Therefore, we inserted only the sensor needle into the anterior chamber (via a pre-loaded 18 G assembly) and placed the infusion needle into the vitreous cavity. This configuration, compared to infusing into the anterior chamber, reduced the risk of fluid leakage under high pressure and helped maintain corneal shape stability, thereby enabling reliable rebound measurements.

One experimental macaque eye was taken from the sterile saline-filled reagent bottle, and the surrounding excess muscle, connective tissue, and fascia were removed. Absorbent paper was used to remove residual fluid from the surface and surrounding area of the eye, keeping the eye relatively dry to facilitate subsequent puncture and implantation of the invasive fluid pressure sensor.

A 24G needle was used to slowly create a puncture at the corneoscleral junction, penetrating the cornea to reach the anterior chamber of the experimental macaque eye. After removing the needle, an 18G needle was carefully inserted into the previously created puncture to enlarge the opening, allowing for the implantation of the invasive fluid pressure sensor. The 18G needle was then withdrawn, and the fluid pressure sensor, enclosed within the needle, was slowly inserted into the puncture site with moderate force to avoid perforating the eye or damaging the sensor probe. Once the sensor needle tip was fully inserted into the anterior chamber and there was no significant leakage from the puncture site, absorbent paper was used to remove residual fluid around the puncture, and cyanoacrylate tissue adhesive was applied to seal the site.

**Fig. 1:**
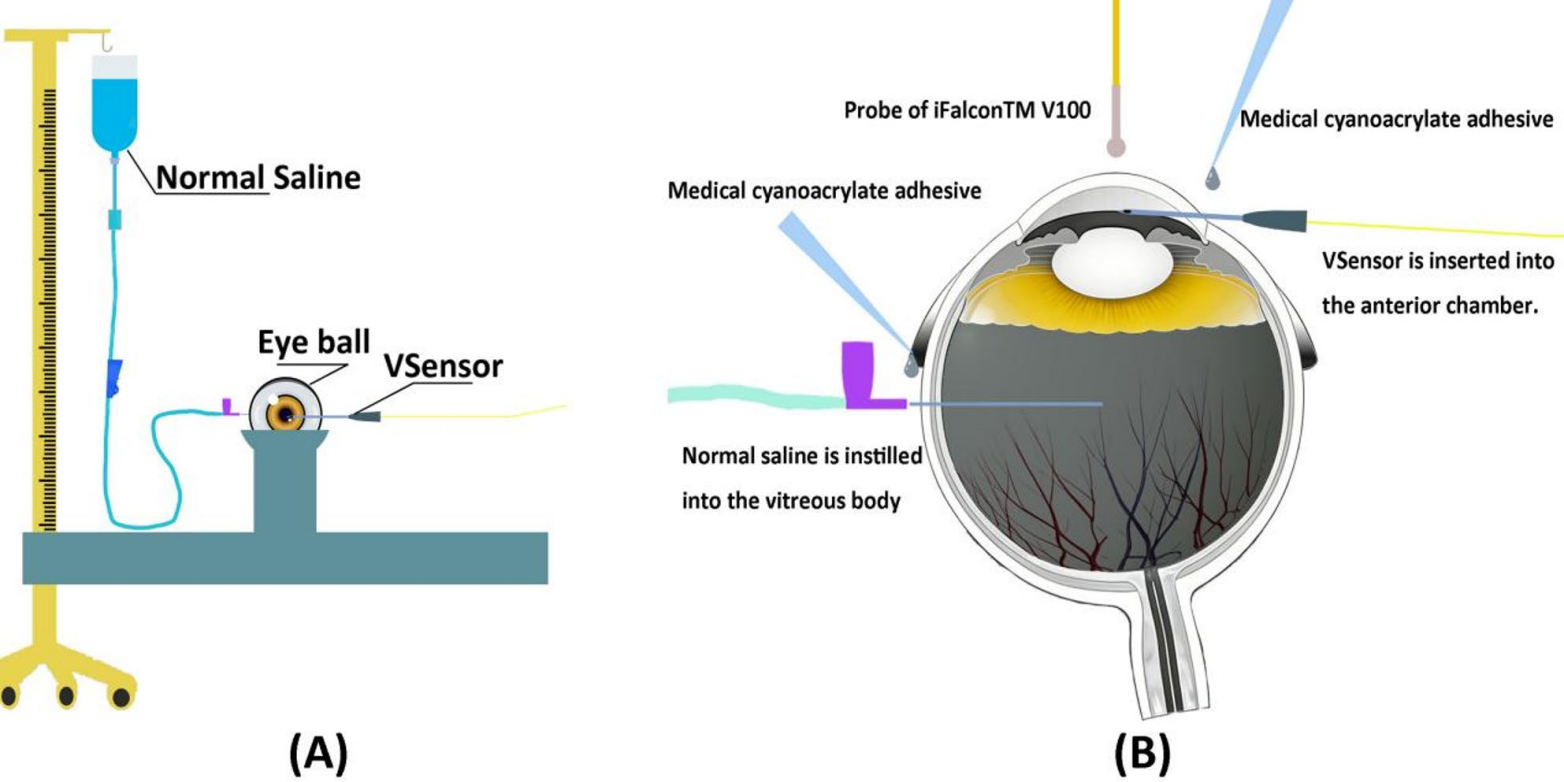
Schematic of the experimental setup: **A** Experimental overview. **B** Needle Insertion Site Diagram

The disposable infusion set was connected to the saline drip bottle, ensuring that the puncture at the top of the bottle was open to the atmosphere. Once the intravenous needle produced a steady, uniform drip, the flow regulator was set to the minimum to stop fluid flow. Next, the intravenous needle was inserted into the vitreous cavity at a position approximately 180° opposite to the corneal puncture site. Cyanoacrylate tissue adhesive was also applied near the scleral puncture site for sealing, and no fluid leakage was observed. As shown in Fig. 1.

IOP was increased stepwise from 5 mmHg to 90 mmHg in increments of 3–5 mmHg by raising the height of the saline drip bottle, while slowly adjusting the flow regulator wheel to maintain a low flow rate. At each target pressure level, the system was allowed to stabilize for 10 s, which was confirmed by real-time monitoring of the invasive pressure sensor. The tonometer was triggered two or three times, and each trigger produced an average of six consecutive readings. Thus, two or three data points were obtained per pressure level. The iFalcon™ V100 rebound tonometer was then triggered remotely via a Bluetooth connection from a computer, without direct hand contact with the device. The tonometer automatically recorded six consecutive readings and displayed their average, which was taken as the IOP at that pressure level. This process was repeated for each pressure step. The operator was blinded to the invasive pressure reading at the moment of triggering, because the computer program simultaneously captured both the tonometer average and the pressure sensor value, displaying them only after the measurement was completed. All measurements were performed by a single operator.

### Experimental platform setup

**Fig. 2:**
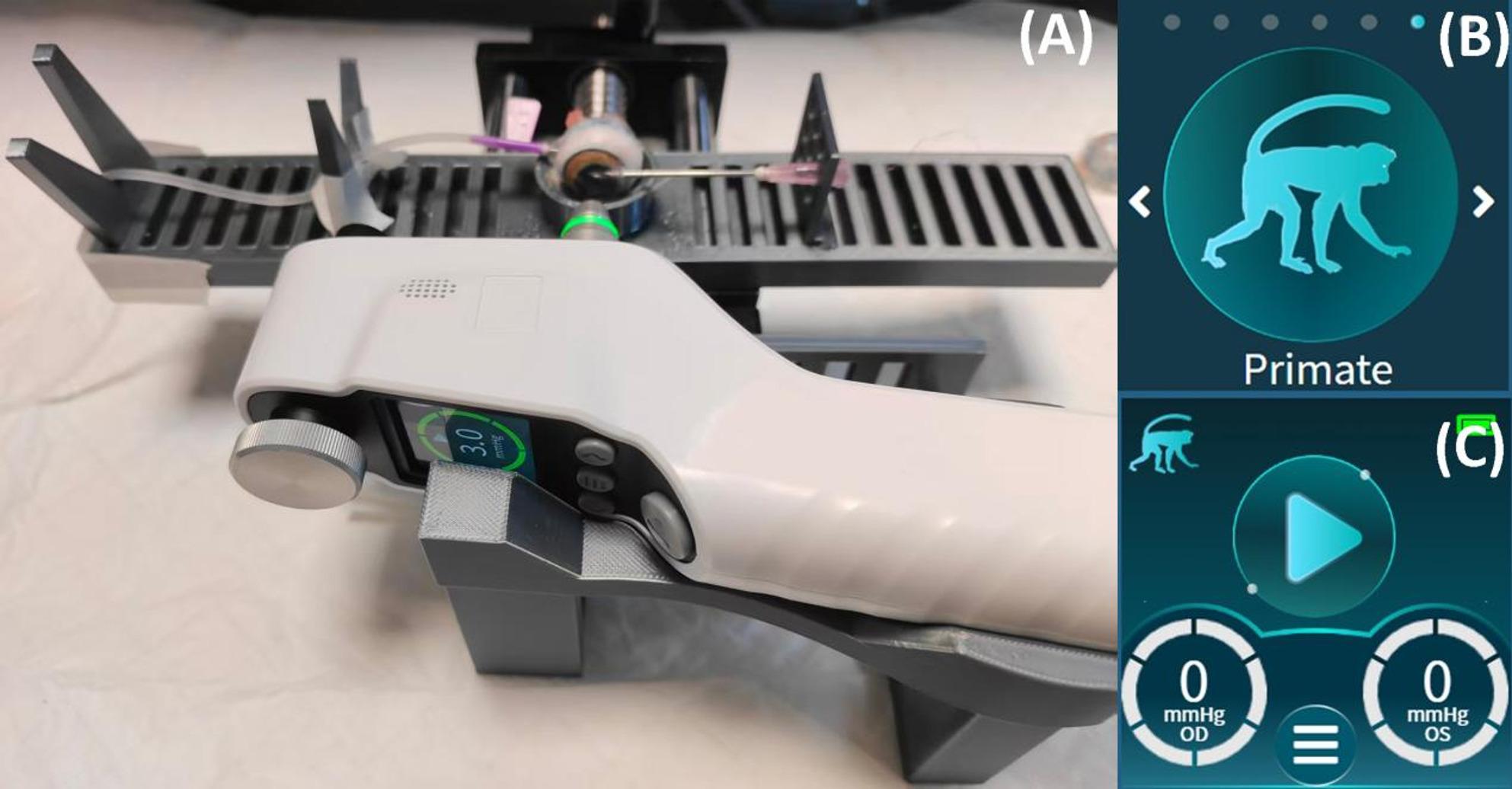
Experimental platform: **A** Actual testing fixture. **B** iFalcon™ V100 macaque-eye mode selection UI. **C** iFalcon™ V100 macaque-eye measurement UI

The ex vivo macaque eye was placed in a 3D-printed support bowl, with the invasive pressure sensor mounting bracket and the disposable infusion set holder positioned on the left and right sides of the bowl, respectively. A dedicated mounting bracket for the iFalcon™ V100 rebound tonometer was placed in front of the support bowl, ensuring that the probe maintained a consistent distance from the corneal center for each measurement and that the relative position between the probe and the cornea remained unchanged. The experimental platform setup is shown in Fig. 2(A), while Fig. 2(B) and Fig. 2(C) display the user interface of the iFalcon™ V100 macaque-eye-specific mode.

### Statistical methods

Linear regression analysis and Bland-Altman agreement analysis were used to evaluate the relationship and agreement between iFalcon™ V100 measurements and invasive pressure sensor readings. Normality of the differences was assessed using Q-Q plots. Descriptive assessment of data distribution was performed. Statistical analyses were conducted using MATLAB (MathWorks, Natick, MA, USA). Linear regression and correlation coefficients were calculated using the built-in functions polyfit and corrcoef. Bland-Altman limits of agreement (LoA) were computed using standard formulas (mean difference ± 1.96 × SD). No custom statistical algorithms were written; all calculations rely on MATLAB’s core mathematical libraries.

The goodness of fit of the linear regression model was evaluated using the coefficient of determination (R²) [24]. For Bland-Altman analysis, the 95% LoA were defined as the mean difference ± 1.96 × standard deviation of the differences [25, 26]. Normality of the differences was assessed using a Q-Q plot.

## Results

### Linear regression analysis

**Fig. 3:**
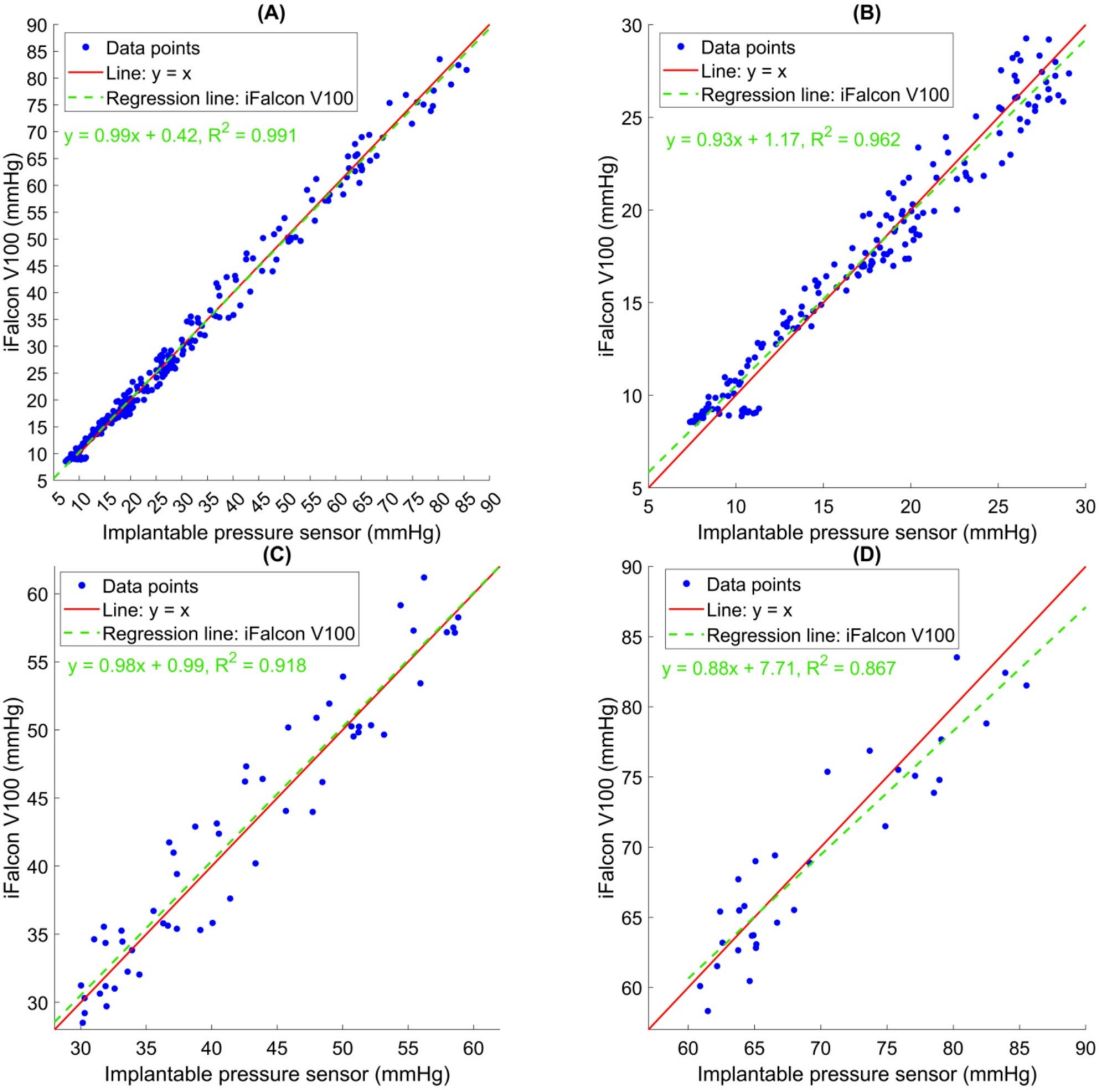
Linear regression analysis: **A** Linear regression of all data points. **B** Linear regression in the 5-30 mmHg range. **C** Linear regression in the 30-60 mmHg range. **D** Linear regression in the 60-90 mmHg range

Through linear regression analysis (least squares method), the relationship between the iFalcon™ V100 rebound tonometer and the invasive pressure sensor was evaluated across the full measurement range. A total of 263 paired data points were collected (Fig. 3A). The regression equation was y = 0.99x + 0.42, with R² = 0.99. This indicates a very strong positive linear correlation over the 5–90 mmHg range.

Although the overall correlation was excellent, examination of the scatter plot revealed differences in data distribution at low, medium, and high IOP levels. Therefore, the data were further divided into three subsets: 5–30 mmHg, 30–60 mmHg, and 60–90 mmHg, and separate regression analyses were performed.

- 5–30 mmHg: The regression line was y = 0.93x + 1.17, R² = 0.96 (Fig. 3B). The high R² value indicates that the two methods maintain a strong linear correlation in the low IOP range.
- 30–60 mmHg: The fitted equation was y = 0.98x + 0.99, R² = 0.92 (Fig. 3C). The correlation remains strong, though slightly weaker than that in the lower range.
- 60–90 mmHg: Regression gave y = 0.88x + 7.71, R² = 0.87 (Fig. 3D). The correlation is relatively lower, and the slope deviates from unity, indicating that the tonometer tends to underestimate IOP at high pressures.

### Agreement analysis

**Fig. 4:**
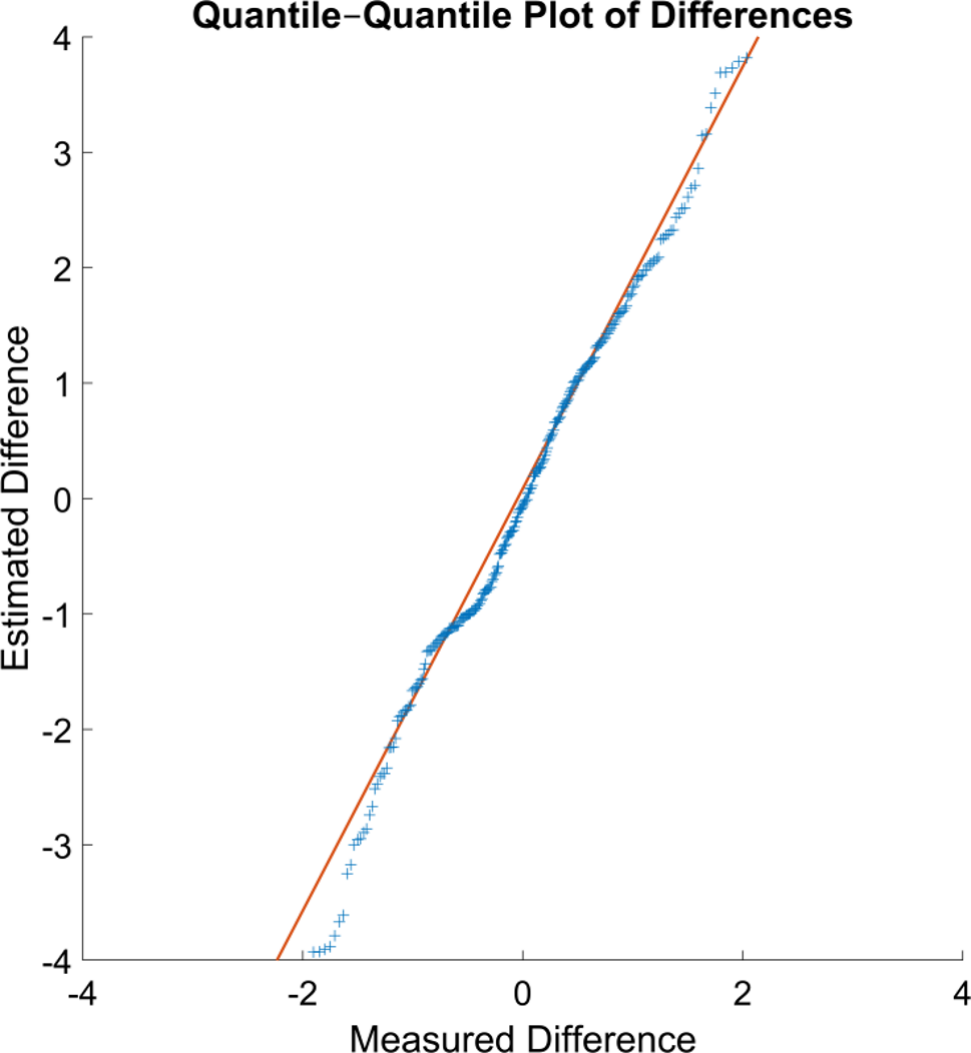
Quantile-quantile plot

A Q-Q plot (Fig. 4) confirmed that the differences between the two methods were approximately normally distributed, satisfying the prerequisite for Bland-Altman analysis.

**Fig. 5:**
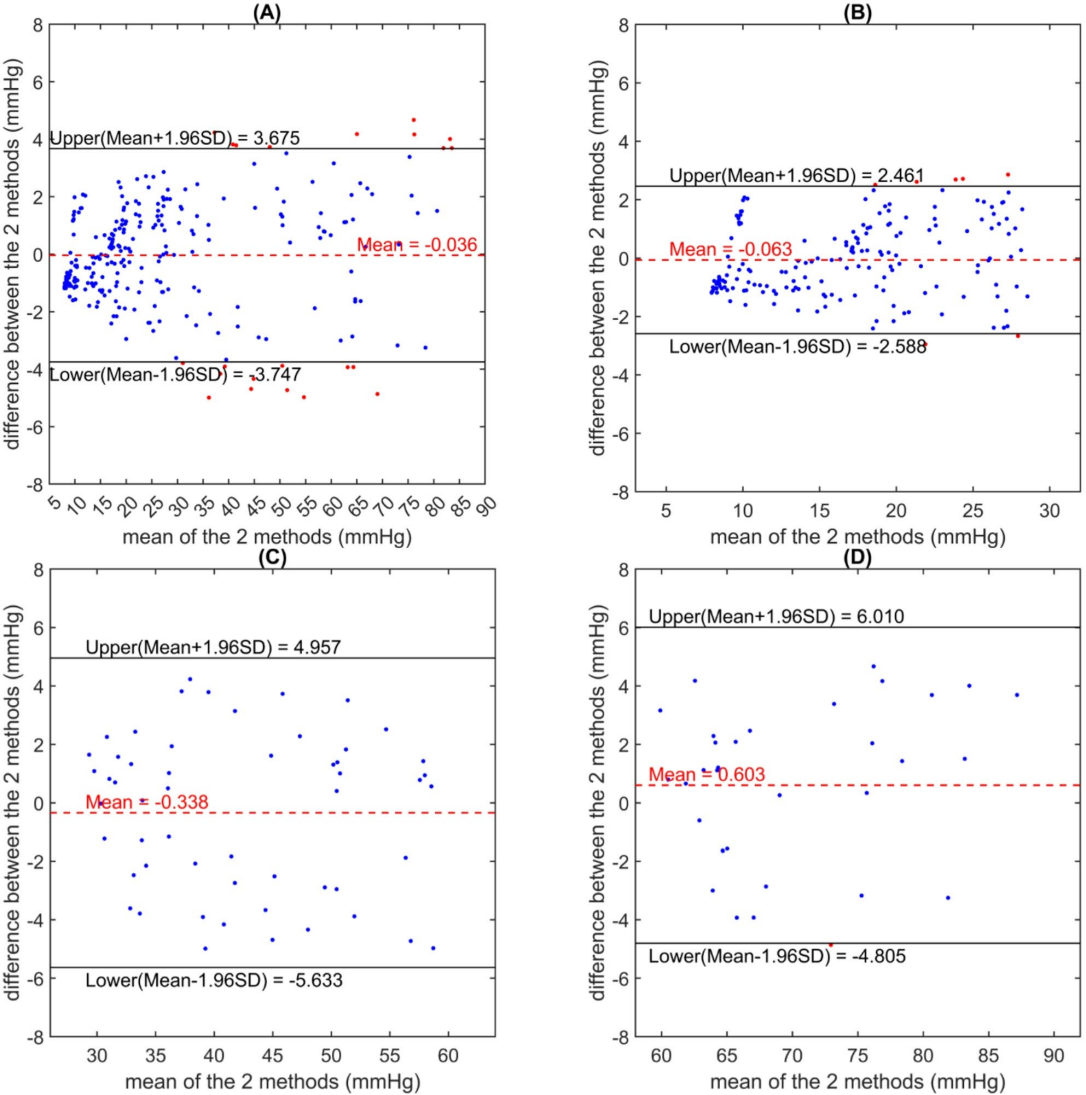
Bland-Altman analysis: **A** Bland-Altman plot for all data points. **B** Bland-Altman plot in the 5-30 mmHg range. **C** Bland-Altman plot in the 30-60 mmHg range. **D** Bland-Altman plot in the 60-90 mmHg range

Overall agreement (5–90 mmHg, 263 points, Fig. 5A):

The mean difference (bias) was − 0.036 mmHg (SD = 1.893 mmHg), with 95% LoA of 3.675 mmHg and − 3.747 mmHg. The narrow confidence intervals indicate limited variability and good agreement across the full pressure range.

Performance across pressure ranges:

To examine potential differences, data were divided into three subsets 5–30 mmHg, 30–60 mmHg, and 60–90 mmHg. The key results are as follows:

In the low, 5–30 mmHg range (176 points), the bias was − 0.063 mmHg (SD = 1.288 mmHg) with 95% LoA of 2.461 mmHg and − 2.588 mmHg. This indicates that the iFalcon™ V100 rebound tonometer has excellent consistency with the measurement results of the invasive pressure sensor within the low IOP range, and meets the high methodological quality standards.

In the moderate range (30–60 mmHg, 54 points), bias was − 0.338 mmHg (SD = 2.702 mmHg) with LoA of 4.958 mmHg and − 5.633 mmHg. Although the confidence interval is slightly wider, it also means that the iFalcon™ V100 rebound tonometer can maintain overall acceptable consistency with the invasive pressure sensor within the moderate IOP range, which is in line with the expectations of the basic experimental method.

In the high range (60–90 mmHg, 33 points), bias was 0.603 mmHg (SD = 2.759 mmHg) with LoA of 6.010 mmHg and − 4.805 mmHg. The iFalcon™ V100 rebound tonometer, within the high IOP range, can be considered to fall within the acceptable range for initial exploratory use.

In summary, the iFalcon™ V100 demonstrated good agreement with the invasive sensor across the entire IOP range, with excellent performance in the clinically most relevant 5–30 mmHg range. The device also maintained acceptable agreement at moderate and high pressures, supporting its utility over a wide spectrum of IOP values.

## Discussion

This study systematically compared measurements from the iFalcon™ V100 and the invasive fluid pressure sensor using two ex vivo macaque eyes from the same animal. The results indicate that the tonometer shows good agreement with the reference sensor within the IOP range, supporting its potential for use in non‑human primate research. However, given the limited sample size, these findings should be considered preliminary. Future studies with more eyes from multiple animals of different ages and sexes are necessary to fully validate the device. Macaque eyes have been used in laser trabecular ablation [27], macular studies [28], and other ophthalmic research, where IOP measurement is required both preoperatively and postoperatively as a fundamental assessment parameter. Therefore, the iFalcon™ V100 holds unique practical value in these contexts. Given the close anatomical and physiological similarities between NHPs and humans, accurate IOP measurement in NHPs is crucial for translational research aimed at developing new glaucoma treatments and surgical techniques for human patients.

The novelty of the iFalcon™ V100 lies in its specialized design and development for IOP measurement in NHPs. In contrast, most commercially available rebound tonometers currently do not offer a dedicated measurement mode for macaques or other NHPs. For example, the Reichert^®^ Tono-Vera^®^ Vet Tonometer currently provides dedicated measurement modes only for dogs, cats, rabbits, and horses [29–31]. Similarly, veterinary rebound tonometers from iCare, such as the TONOVET Pro [32] and TONOVET Plus [33], also have built-in measurement modes limited to common animals like dogs, cats, rabbits, and horses. Some studies have not been strictly limited by the device’s preset measurement modes. For example, Mills et al. used the Tono-Vera^®^ Vetto measure IOP in pig eyes [34], and Yong Yiet al. also used the Tonovet to measure IOP in *Macaca thibetana* [35]. Although these studies reported relatively good measurement results, using eye modes designed for other species to test non-preset groups still presents certain limitations. Overall, current research on IOP in macaques still lacks systematic validation data obtained using measurement modes specifically designed for this species.

Early studies conducted by the Bito team measured IOP using applanation tonometry under local anesthesia [12]. Literature reports indicate that in generally healthy macaques without ocular diseases, the average IOP is approximately 14.9 ± 2.1 mmHg, with a range of 10–21 mmHg [12]. The sample size in that study was just over 100 subjects, making the data set relatively limited. Subsequently, the Pasquale team conducted a systematic survey of 722 macaques [36], significantly increasing the sample size and enhancing the statistical reliability of the results. Their study reported that the average IOP in healthy macaques was 13.0 ± 4.3 mmHg, with a range of 2–27 mmHg [36]. In that study, the IOP of suspected glaucoma macaques were 22 mmHg and 20 mmHg in the right and left eyes, respectively [36]. In addition, Xu Jia et al. reported that suspected glaucoma macaques had an average IOP of 28.17 ± 2.14 mmHg, with a range of 26–32 mmHg [37]. Overall, previous research indicates that IOP values in macaques, both healthy and those with suspected glaucoma, typically fall within the range of approximately 10–40 mmHg, which is well covered by the iFalcon™ V100’s measurement range. The present ex vivo study, using eyes from a healthy animal, showed that the tonometer provides accurate IOP measurements (mean difference = -0.063 mmHg, LoA = -2.59 to 2.46 mmHg) within the 5–30 mmHg range. However, because our tested eyes had no known ocular pathology, we cannot directly extrapolate these findings to diseased eyes with altered corneal or scleral biomechanics. Further studies using eyes with confirmed glaucoma or other abnormalities are needed to assess device performance under pathological conditions. Even at higher pressures (up to 90 mmHg), agreement remained acceptable, suggesting that the device maintains reasonable performance over a wide pressure range, although the clinical relevance of such extreme pressures in macaques is limited.

### Limitations

Several limitations should be acknowledged. First, the small sample size—only two eyes from a single 12-year-old male rhesus macaque—severely limits the generalizability of our findings. While this was due to the significant difficulty and ethical constraints in obtaining non-human primate eyes, it also means that we could not account for potential effects of age, sex, or individual biological variability. Therefore, our work should be regarded as a feasibility study that provides initial evidence of the tonometer’s performance. Confirmatory studies with more eyes from multiple animals of different ages and sexes are essential before the device can be recommended for routine use in NHPs research.

Second, the device frequently failed to provide valid measurements when IOP fell below 7 mmHg because the cornea was judged “too soft”. In the high IOP range, the device tended to slightly underestimate the true pressure. Multiple studies have reported reduced accuracy and reproducibility when measuring at either low or high IOP extremes [38, 39], indicating that this is not a unique limitation of our device. In clinical or research practice, such a low IOP already suggests potential ocular abnormality (healthy macaque IOP is typically ≥ 10 mmHg), so the inability to obtain a reading can serve as a qualitative sign. However, for investigators who require precise quantitative measurements in the hypotony range, this device has limited applicability and alternative methods should be considered. For high IOP levels (e.g., above 50 mmHg), although the device does not provide extremely accurate data, the readings still offer valid reference value for veterinary practitioners to identify ocular hypertension.

Third, pressurization was achieved by infusing saline into the vitreous cavity rather than into the anterior chamber. Although this configuration reduced the risk of corneal deformation that would have occurred if both the sensor needle and the infusion needle were placed in the limited anterior chamber space, it may still have introduced small deviations from true physiological IOP elevation. The sensor needle itself remained in the anterior chamber, but its presence could have slightly altered corneal shape and, consequently, IOP measurements. It should be noted that, during the experimental procedures, the researchers carefully positioned the sensor needle in the posterior part of the anterior chamber during insertion, minimizing corneal deformation and potential interference of the needle tip with the rebound tonometry measurements. Nevertheless, any residual corneal deformation, however minor, could still affect the IOP readings [40]. We chose this compromise because inserting two needles into the anterior chamber would have caused severe corneal distortion and compromised the rebound measurements. The potential influence of this setup on the results should be recognized.

Fourth, this study used ex vivo non-human primate eyes and did not conduct validation in live macaques. This design allowed us to evaluate measurement accuracy under highly controlled conditions, avoiding potential interference from extraocular muscle tone, blood perfusion, and neural reflexes. However, the ex vivo model cannot fully replicate the complex physiological conditions of the in vivo eye. Therefore, the lack of in vivo testing remains a limitation, and future work is needed to further validate the device’s applicability under real physiological conditions in live NHPs or clinical settings. In addition, some studies have indicated that normal saline use may increase corneal damage [41].

Although this study has certain limitations, these shortcomings do not compromise the overall interpretation of the results. Overall, the iFalcon™ V100 demonstrates significant medical and scientific value both in the measurement of IOP in NHPs and in the advancement of rebound tonometry technology.

## Conclusions

This study is the first to systematically evaluate the IOP measurement performance of the iFalcon™ V100 in ex vivo non-human primate eyes. The results demonstrate good correlation and agreement between the iFalcon™ V100 readings and the invasive pressure sensor across a wide pressure range. While the sample size is limited, these preliminary findings support the potential of this species-specific measurement mode for IOP assessment in non-human primate research. Further studies involving more eyes and in vivo conditions are needed to confirm its reliability and repeatability.

## Data Availability

The data are available from the corresponding author upon reasonable request.
